# Bilateral Pyosalpinx Due to a 16-Year-Old Intrauterine Device Presented as Appendicitis

**DOI:** 10.7759/cureus.48257

**Published:** 2023-11-04

**Authors:** Mohammad Othman

**Affiliations:** 1 Clinical Sciences Department, Fakeeh College for Medical Sciences, Jeddah, SAU

**Keywords:** bilateral salpingectomy, uncomplicated appendicitis, intrauterine device, pelvic inflammatory disease, pyosalpinx

## Abstract

Pyosalpinx is the collection of pus in the fallopian tube. Pyosalpinx usually follows pelvic inflammatory disease, sexually transmitted disease, or rarely non-sexually transmitted infection. This is the first-ever report of bilateral pyosalpinx due to intrauterine device in situ for the past 16 years, which presented as appendicitis. Pyosalpinx should be considered in female patients with lower abdominal pain.

## Introduction

Pyosalpinx usually affects sexually active women aged 20 to 40 years old [1,2]. These women always give a history of pelvic inflammatory disease (PID) [2-4]. These women suffer from fever and chronic pain in the lower abdomen [2,5]. The pain could be with sex, with menses, or even all the time [2,5,6]. With time, this pelvic inflammation will result in the accumulation of pus in the fallopian tubes and the occurrence of pyosalpinx [7]. Many doctors think pyosalpinx is mainly associated with sexually transmitted diseases, especially chlamydia and gonorrhea [2,3,7]. In fact, developing pyosalpinx due to nonsexual bacteria without PID is rare and seen in elderly women and non-sexually active little virgin girls [4,7]. On top of that, developing pyosalpinx due to an intrauterine contraceptive device (IUCD) is extremely rare [2,6,8]. This paper reports on a patient with bilateral pyosalpinx due to an old IUCD.

## Case presentation

**Figure 1:**
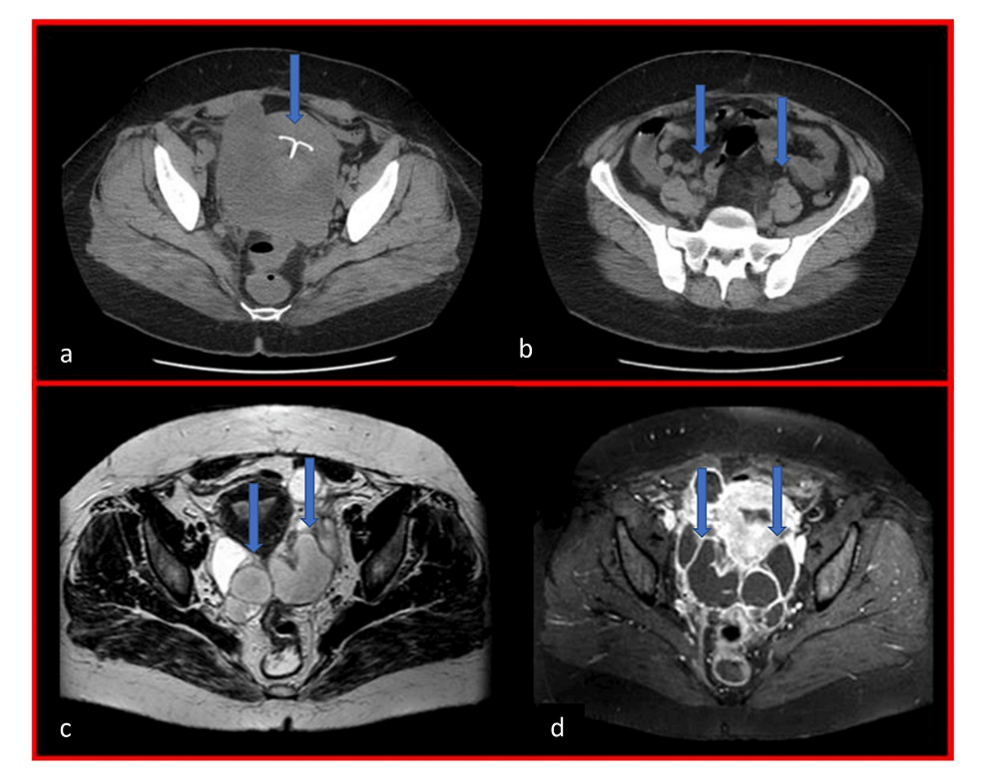
CT scan transverse sections showing IUCD, uterus, and overdistended tubes (a) Arrow showing intrauterine contraceptive device (IUCD) inside the uterus. (b-d) Arrows pointing to overdistended tubes on both sides.

**Figure 2:**
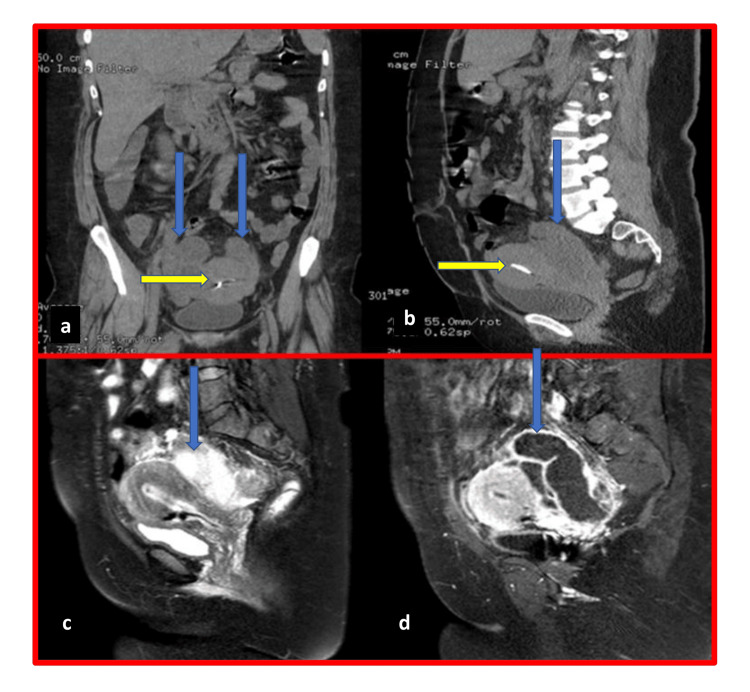
CT scan coronal sections showing IUCD, uterus, and overdistended tubes (a and b) Intrauterine contraceptive device (IUCD) inside the uterus (yellow arrow) and over distended tubes (blue arrows). (c and d) Over distended tubes (blue arrows).

A 38-year-old woman was admitted by the surgical team for confirmation of appendicitis and possible removal. The patient gave a history of IUCD insertion 16 years ago and a history of high fever on and off for 10 days. The patient was referred to the gynecologist to check on the IUCD. This patient was found to have copper IUCD inserted 16 years ago, and she and her husband do not want any children. Noticeably, her fever and lower abdominal pain disappeared completely every time after she took two tablets of paracetamol in the past 10 days. On abdominal ultrasound, no abnormality was detected, and IUCD seems intact. On bimanual examination, fullness was felt in both adnexa and severe tenderness was noticed in the right side. The patient was sent for a CT scan. In the CT scan, both tubes were seen twisted full, extremely wide, and massive (Figures 1, 2).

In view of contrast-enhanced computed tomography (CECT) findings, the decision of abdominal exploration was taken with possible bilateral salpingectomy, removal of the IUCD, and dilatation and curettage (D&C). The patient and her husband refused any possibility of hysterectomy with bilateral salpingectomy.

**Figure 3:**
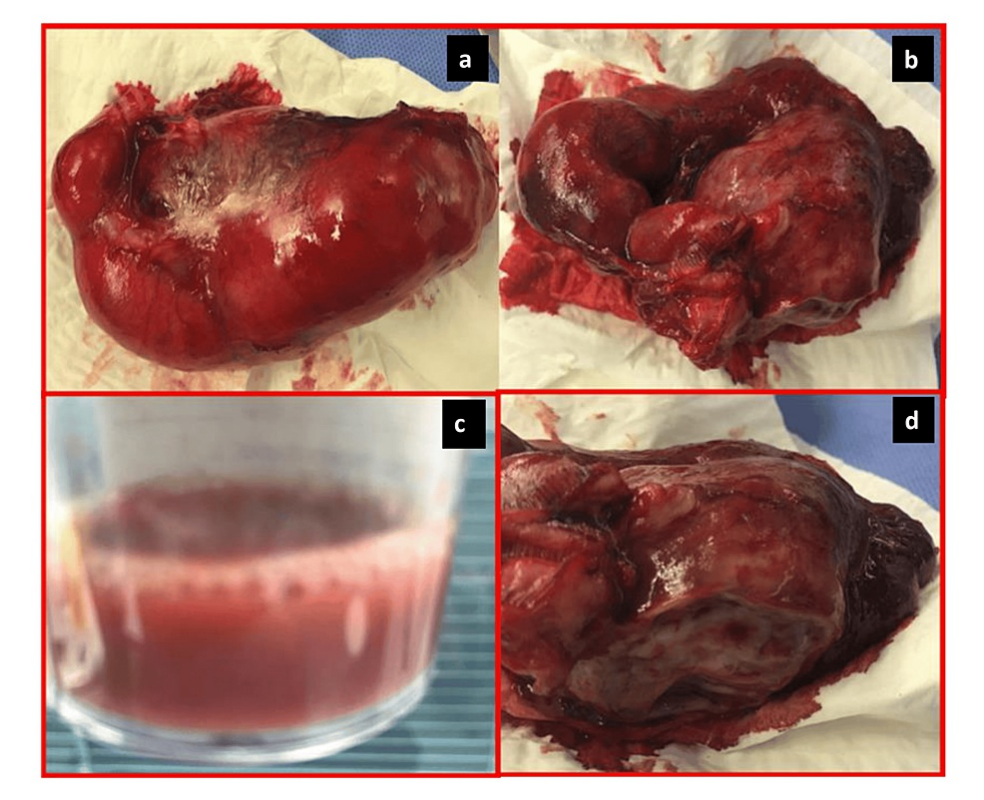
Tubes after removal Left tube (a), right tube (b and d), and the amount of pus in both tubes (c).

During the operation, the uterus was normal in size. Both tubes were edematous, extremely wide, and convoluted around some parts of the small intestine. The right tube was convoluted around the small bowel with many adhesions between their walls. Both tubes were removed completely and found to be filled with pus of approximately 275 cc (Figure 3). IUCD was removed and D&C was done smoothly.

The patient's stay in the hospital was unremarkable. She was discharged after three days on oral antibiotics. She was seen after two weeks in good condition.

## Discussion

Pyosalpinx is a devastating diagnosis because there will be an affection on the fertility of the patient [2,5,8]. The treatment mostly is salpingectomy but sometimes can be conservative by intravenous (IV) antibiotics or laparoscopic aspiration [3,5,6]. Most pyosalpinx cases are sexually transmitted, still, several case reports of non-sexually active virgins and elderly have been reported. This is mainly due to severe vaginitis leading subsequently to salpingitis and usually, these cases are accompanied by decreased immunity diseases like diabetes mellitus [4,7,8]. Risk factors for PID and hence pyosalpinx include obesity, poor hygiene, diabetes, bacterial vaginosis, cervical surgical instrumentation, sexual abuse, multiple sexual partners, previous history of PID, and appendicitis [2,5,6]. Moreover, urogenital malformations and Hirschsprung’s disease have been reported to be present in association with pyosalpinx [1,8].

Pyosalpinx may present with symptoms or remain silent. The absence of specific symptoms and conclusive signs during the physical examination may delay a proper diagnosis [2]. The most common presenting symptom is lower abdominal pain and fever seen only in 50% of women with pyosalpinx [3,6]. Other symptoms may include chills, nausea, vaginal discharge, and abnormal vaginal bleeding [1,5]. On examination, patients show tenderness over the adnexal region with or without guarding or rebound tenderness [1-3,5]. This was similar to our patient who had rebound tenderness and guarding on the right side with pain in the lower abdomen, which led to the first diagnosis of appendicitis.

The best imaging modality in the case of pyosalpinx is transvaginal ultrasound, which was not done in this patient due to extreme tenderness. MRI and CT scans help to give the proper diagnosis [4,5,8]. In this patient, a CT scan differentiated the diagnosis for the patient from appendicitis to pyosalpinx and even showed the possible culprit, which is the old in situ IUCD. IUCD is not very uncommonly seen as a cause for PID but is still extremely rare to be the cause of bilateral pyosalpinx [1,6,8]. On reviewing the literature, there is only one single case that reported pyosalpinx on the left side due to a seven-year-old IUCD that migrated to the tube [1]. This case is the first-ever reported case of IUCD in situ for 16 years to cause bilateral pyosalpinx.

## Conclusions

Pyosalpinx is the collection of pus in the fallopian tube. It is usually following PID, sexually transmitted disease, or rarely non-sexually pelvic infection. This is a report of bilateral pyosalpinx in a patient with IUCD in situ for 16 years, which presented as appendicitis. Differential diagnosis of pyosalpinx must be considered in female patients with lower abdominal pain.
